# Cost-effectiveness of village health worker-led integrated community case management (iCCM) versus health facility based management for childhood illnesses in rural southwestern Uganda

**DOI:** 10.1186/s12936-024-04962-7

**Published:** 2024-05-15

**Authors:** Edgar Mulogo, Moses Ntaro, Andrew Wesuta, Jane Namusisi, Peter Kawungezi, Vincent Batwala, Michael Matte

**Affiliations:** 1 Department of Community Health, Mbarara University of Science and Technology, PO Box 1410, Mbarara, Uganda; 2 Bugoye Community Health Collaboration, P.O. Box 149, Kasese, Uganda; 3 Department of Pediatrics, Mbarara Regional Referral Hospital, P.O. Box 40, Mbarara, Uganda; 4 Directorate of Research and Graduate Training, Mbarara University of Science and Technology, P.O. Box 1410, Mbarara, Uganda

**Keywords:** Cost effectiveness, Village health workers, Integrated community case management, Facility based services, Cases treated, Uganda

## Abstract

**Background:**

In Uganda, village health workers (VHWs) manage childhood illness under the integrated community case management (iCCM) strategy. Care is provided for malaria, pneumonia, and diarrhoea in a community setting. Currently, there is limited evidence on the cost-effectiveness of iCCM in comparison to health facility-based management for childhood illnesses. This study examined the cost-effectiveness of the management of childhood illness using the VHW-led iCCM against health facility-based services in rural south-western Uganda.

**Methods:**

Data on the costs and effectiveness of VHW-led iCCM versus health facility-based services for the management of childhood illness was collected in one sub-county in rural southwestern Uganda. Costing was performed using the ingredients approach. Effectiveness was measured as the number of under-five children appropriately treated. The Incremental Cost-Effectiveness Ratio (ICER) was calculated from the provider perspective.

**Results:**

Based on the decision model for this study, the cost for 100 children treated was US$628.27 under the VHW led iCCM and US$87.19 for the health facility based services, while the effectiveness was 77 and 71 children treated for VHW led iCCM and health facility-based services, respectively. An ICER of US$6.67 per under five-year child treated appropriately for malaria, pneumonia and diarrhoea was derived for the provider perspective.

**Conclusion:**

The health facility based services are less costly when compared to the VHW led iCCM per child treated appropriately. The VHW led iCCM was however more effective with regard to the number of children treated appropriately for malaria, pneumonia and diarrhoea. Considering the public health expenditure per capita for Uganda as the willingness to pay threshold, VHW led iCCM is a cost-effective strategy. VHW led iCCM should, therefore, be enhanced and sustained as an option to complement the health facility-based services for treatment of childhood illness in rural contexts.

## Background

In 2019, diarrhoea, malaria, and pneumonia accounted for almost 30% of all deaths worldwide in children under the age of five [1]. Each year, approximately 5 million cases of malaria, pneumonia, and diarrhoea in the community are treated inadequately, slowly, or not at all [2]. Additionally, it has been reported that these childhood ailments claim the lives of 1.8 million children under the age of five year [3]. Community health structures are widely acknowledged as an essential platform for delivering Primary Health Care (PHC) services globally, and their accomplishment is closely associated with the attainment of Universal Health Coverage and the Sustainable Development Goals (SDGs) [4]. It has been demonstrated that PHC’s integrated programming increases the provision of vital community services, improves the coverage of vital interventions, and improves health outcomes [5]. A large number of children who reside in remote locations do not receive medical care within the critical 24-h window [2]. However, since 2012, the World Health Organization (WHO) and (United Nations Children’s Fund) UNICEF have recommended iCCM of childhood illnesses as a core component of the health facility based integrated management of childhood illness (IMCI) [5].

Many developing nations have implemented iCCM to increase access to treatment for these illnesses [6, 7]. iCCM is a component of a larger VHW strategy, wherein VHWs treat childhood killer diseases within 24 h of symptom onset in order to save lives [2]. Trained community health workers (VHWs) treat paediatric malaria, pneumonia, and diarrhoea under the iCCM approach, typically in a community setting as opposed to a medical facility [2, 8]. Consequently, iCCM lowers out-of-pocket costs, improves access to care, and brings treatment closer to home [2, 5]. Using a clinical algorithm, the VHWs diagnose and treat malaria, pneumonia, and diarrhoea. This involves giving any child who presents with a subjective fever a rapid diagnostic test (RDT) for malaria and interpreting the test's results [8]. By improving access to timely care, iCCM may be able to lower morbidity and death rates [9].

VHWs from the national programme in this area have been providing iCCM care since 2013, with financial and operational support from a long-standing collaboration with Mbarara University of Science and Technology (Mbarara, Uganda) and the Massachusetts General Hospital (Boston, Massachusetts, USA). The study was conducted in the rural, mountainous Bugoye sub-county in Kasese District (on the western border of Uganda with the Democratic Republic of Congo) [9]. While the Bugoye sub-county’s network of Health Centres Level II–III provides facility-based management of paediatric illnesses. Constraints on human resources and accessibility variations have an impact on the care services offered at the health facility level. Another factor limiting access to facility-based care is lost opportunities in healthcare facilities. The Ministry of Health planned to use a combination of high impact, low cost interventions, like iCCM, in conjunction with the healthcare system to close the significant treatment gap [10]. A sizeable amount of family budgets and government spending goes toward health services. As a result, governments are actively looking for methods to reduce expenses, increase efficiency, and obtain more funding [11].

Research on VHW-led iCCM in southwest Uganda has concentrated on performance, service coverage, referral systems, care quality, utilization, and satisfaction [8, 9, 12–14]. There is currently little data in Uganda on how much more cost effective iCCM is when compared to facility-based care for paediatric ailments”. Given the competing priorities of governments and the limited funding available, it is necessary to assess the costs, costs-effectiveness, and affordability of such programmes in order to mobilize the necessary resources to overcome these challenges at the Information about the economic assessment of health care programmes in Uganda is scarce. Data from economic assessments is used to inform health sector intervention planning. In a rural sub-county in southwest Uganda, this analysis contrasts the cost-effectiveness of using the VHW led iCCM for managing childhood illness with services provided by health facilities.

## Methods

### Design and setting

A cost-effectiveness analysis study was carried out using data gathered between February and May 2023, comparing VHW led iCCM with health facility = based treatment for malaria, pneumonia, and diarrhoea in children under five. The decision making process was organized using decision analytic modelling. With an average household size of 5.6, the Bugoye sub-county is home to just over 45,000 people. The most common childhood illnesses are respiratory infections and malaria. Under the national programme, 22 villages in the sub-county surrounding Bugoye Health Centre level III were served by VHWs trained and equipped with medical supplies to perform iCCM services. Each village has four to five VHWs who make up the VHW teams, which are chosen by the village community. There are eight health facilities in the sub-county in total: two level III health centres, six level II facilities, and one private-not-for-profit (PNFP) facility. The sub-county's sole public level III medical facility is called Bugoye Health Centre.

### VHW led iCCM

The VHW team is comprehensively trained to identify, treat, and/or refer children under five who have diarrhoea, pneumonia, or malaria. First, VHWs review their responsibilities during a basic 3-day training that is standardized by the Ugandan Ministry of Health (MoH). This was followed with a 6-day training on implementing iCCM services [15]. By improving access to timely care, iCCM may be able to lower morbidity and death rates [9]. The VHWs assess each patient's need for care using the iCCM protocol, also known as the “Sick Child Job Aid.” They are prepared to diagnose patients with subjective fever using rapid diagnostic tests (RDT) for malaria. Clinical history is used to diagnose diarrhoea, while age-based respiratory rate cut-offs are used to diagnose pneumonia. VHWs provide initial assessment, referral or accompaniment to a health facility, as well as pre-referral treatment for certain conditions, for patients exhibiting “danger signs,” or indications of a serious illness [16]. The programme funding covers medications for the treatment of iCCM.

Rapid diagnostic tests (RDTs) for malaria, respiratory timers, artemether/lumefantrine (20 mg/120 mg tablets), amoxicillin (125 mg dispersible tablets), low osmolarity ORS, zinc (20 mg tablets), and rectal artesunate (50 mg) are all given out free of charge in iCCM kits to the trained VHWs. Cotton wool, gloves, medicine boxes, stock cards, and methylated spirit are among the additional supplies offered. VHWs are provided with incentives like raincoats, umbrellas, gumboots, solar lights, and hoes as a means of encouraging them [17].

To guarantee that they are always providing appropriate under-five febrile disease management, the VHW are routinely supervised to reinforce their knowledge and skills. Every patient that a VHW evaluates is entered into a “Sick Patient Register.” Each month, these Sick Patient Registers are turned in, and the results are added together to produce a “Monthly Report” for the entire programme. One of the study’s data sources was the monthly reports and filed registers [16].

### Health facility based service

A nursing assistant welcomes and registers the under-five children and their caregiver when they arrive at the out-patient department of a primary care facility (HC II and HC Level III). While waiting to see the clinician, an enrolled nurse offers group health education. After that, the caregiver and child are taken to see a clinician for an evaluation. Malaria diagnostic tests are carried out in the facility laboratory, which is staffed by a technician or assistant. Clinicians treat children under five years old in accordance with Ugandan government regulations. Clinical guidelines pertaining to diarrhoea, pneumonia, and malaria. Next, a drug dispenser at the medical facility gives out drugs.

### Data collection and analysis

Data was gathered using records from the VHW-led iCCM programme and a health facility. Financial accounts and reports were reviewed for cost data where necessary. While reviewing the VHW “Sick Patient Register” and health management information system records (HMIS) for effectiveness data. All data gathered was based on implementation in the calendar year 2019.

## Costs

### VHW led iCCM in Bugoye sub-county

A cost analysis was carried out using the “ingredients” approach and taking into account economic costs from the perspective of a health care provider. Each strategy’s resources (type, quantity, and unit price) were recorded. The cost of each resource was determined by multiplying the quantity by the unit cost, and the total cost was determined by adding all of the individual costs together [17, 18]. The cost data collected was adjusted to reflect costs for the analysis period. The costs were expressed in US dollars in 2023 at a rate of UGX 3700 per US dollar. The costs were expressed in 2023 US Dollars at an exchange rate of UGX 3700 per US Dollar (US$) [19].

Capital costs associated with initial training were annualized at a 3% discount rate. Quarterly refresher meetings, management salaries, drug and other supplies (medicines for malaria, pneumonia, and diarrhoea, rapid diagnostic tests, respiratory timer, medicine box, safety box, gloves, cotton wool, methylated spirit, and stock cards, iCCM register) were among the recurring costs. Raincoats, umbrellas, gumboots, solar lights, and hoes were among the incentives [17].

### Health facility based services

The facility-based services’ costs were derived from a study on the cost of treating childhood malaria, diarrhoea, and pneumonia at health facilities in rural Mozambique and Uganda [20].

### Effectiveness

Data on effectiveness (outcome) were collected for the year 2019. The number of children under the age of five who received appropriate treatment for malaria, pneumonia, and diarrhoea was used as a measure of effectiveness, i.e. an outcome measure. A detailed description of the definition of appropriately treated children can be found elsewhere. Data on effectiveness (outcome) were collected for the year 2019. The number of children under the age of five who received appropriate treatment for malaria, pneumonia, and diarrhoea was used as a measure of effectiveness, i.e. an outcome measure. A detailed description of the definition of children “treated appropriately” can be found elsewhere [12, 17]. The effectiveness data were gathered from VHW records in 22 villages and 8 health facilities in Bugoye sub-county.

### Cost effectiveness

**Fig. 1:**
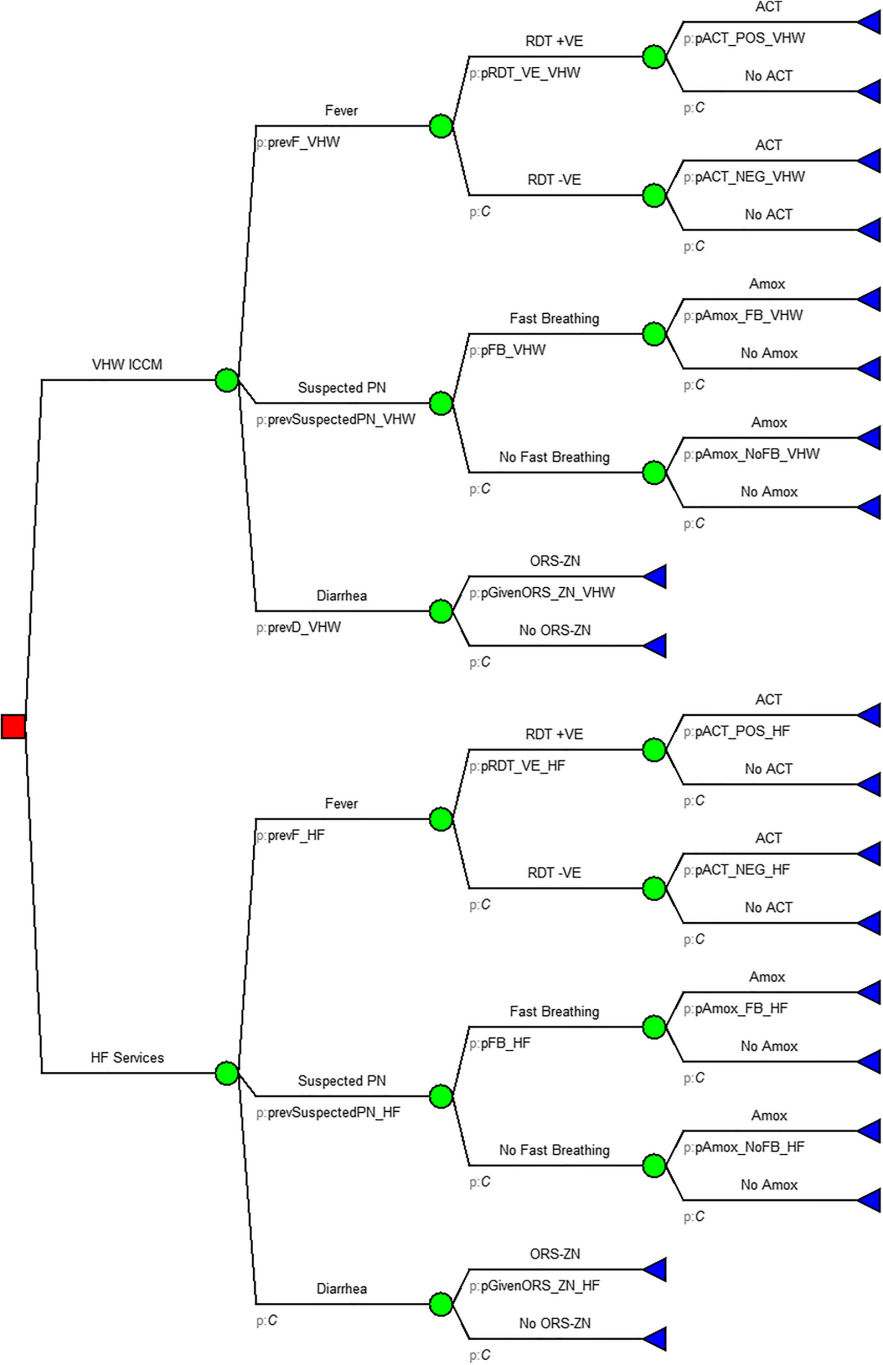
Decision analysis model

Amua software v0.3.0 was used to perform a cost-effectiveness analysis using a decision model [21]. The probabilities used in the tree were derived from primary data from VHW records in 22 villages and 8 health facilities in Bugoye sub-county, as well as related studies on the management of malaria, pneumonia, and diarrhoea in children under the age of five [12, 17, 22, 23]. Figure 1 depicts the decision tree model that was used.

The cost of treating 100 children appropriately under each arm was calculated. An incremental cost effectiveness ratio (ICER) was calculated to assess the additional cost per child treated by the VHW-led iCCM. The ICER is defined as the difference in costs incurred by switching from one intervention to another divided by the difference in outcomes/effectiveness from switching. This ratio was used to calculate the additional cost of producing one additional outcome/effectiveness measure. The willingness to pay (WTP) threshold from a provider perspective was used to determine whether the VHW led iCCM is cost effective. The WTP was established as Uganda's public health expenditure per capita of US Dollar (US$) 21 [24].

### Sensitivity analysis

A one-way sensitivity analysis was performed, taking into account that the proportion of children receiving appropriate treatment for malaria, pneumonia, and diarrhoea was likely to change over time. The impact on the ICER was observed.

## Results

### Costs

Based on the decision modelling for this study, the cost for 100 appropriately treated children was US$628.27 for VHW-led iCCM and US$87.19 for health-care facility-based services. Table 1 shows the costs associated with strategy, broken down by disease condition. Table 1 Costs associated with VHW-led iCCM and health-care facility-based services for 100 children treated in 2023 (US dollars). The unit cost per child treated appropriately for the VHW led iCCM and health facility based strategies was US$6.28 and US$ 0.87, respectively.

**Table 1:** Costs associated with VHW-led iCCM and health-care facility-based services for 100 children treated in 2023 (US dollars)

Cost category	VHW led iCCM (US$) Strategy		
	Malaria	Pneumonia	Diarrhea
Capital			
Training	16.81	6.42	3.72
Recurrent			
Quarterly meetings	31.35	11.98	6.93
Drugs and other supplies	742.18	78.07	107.91
Management salaries	502.54	192.08	111.11
Incentives	48.11	18.37	10.65
Field supervision	26.84	10.26	5.93
Sub-total recurrent costs	1351.01	310.76	249.22
Total cost for 100 children	1367.83	317.19	246.25
Unit cost per child treated	13.67	3.17	2.46
Health facility based services^α^ US$ (range)			
Total cost for 100 children	83.3 (83.3–83.3)	142.8 (80–150)	71.4 (30–90)
Unit cost per child treated	0.83 (0.83–0.83)	1.42 (0.8–1.5)	0.71 (0.3–0.9)

^α^Batura et al. cost estimates[20]

### Cost effectiveness and incremental cost effectiveness ratio (ICER)

The number of children treated appropriately was 77 for the VHW-led iCCM strategy and 71 for the health-care facility-based strategy, respectively. Table 2 shows the calculated cost per 100 appropriately treated children for both strategies. The incremental cost effectiveness ratio is 6.67 US dollars.

**Table 2:** Incremental cost effectiveness ratio per child treated

Strategy	Cost (US$)	Incremental cost (US$)	Effectiveness (children treated)	Incremental effectiveness (children treated)	Cost/ effectiveness (US$/children treated)	ICER^a^
VHW-led iCCM	628. 27	–	77	–	8.16	–
Health facility services	87.19	541.08	71	6	1.22	6.67

^a^Incremental cost effectiveness ratio

### Sensitivity analysis

The proportion of children treated appropriately for fever, pneumonia, and diarrhoea did not affect the cost effectiveness of the VHW-led iCCM strategy.

## Discussion

According to this study, the VHW-led iCCM strategy is more effective at treating children appropriately. The costs for each child treated appropriately, however, were higher for the VHW-led iCCM strategy than for health-care facility-based services. This is in contrast to findings elsewhere that reported higher costs for facility based integrated management of childhood illness (IMCI) in contrast to iCCM [25]. The higher costs in the VHW led iCCM strategy have been attributed to recurrent costs associated with management of the programme [17]. It is important to note, however, that in order for iCCM programmes to be cost-effective and affordable, they must be well-utilized, with programme management and supervision organized to minimize costs while ensuring quality of care. iCCM programmes will not always be inexpensive, especially in small, remote villages where supervision and supply challenges are greater [6].

The cost per child treated appropriately under the VHW led iCCM for malaria is comparable to that reported in another study [26]. The costs per child treated appropriately for the different illness were on average is within the same range with those reported for iCCM programmes elsewhere; US$ 1.54–17.54, US$ 1.12–12.94, and US$ 0.38–13.71 for malaria, pneumonia and diarrhoea, respectively [6, 27]. The variations observed in costs between different iCCM programmes are can be attributed to recurrent costs such as training, incentives provided and supervision among others. The cost per child treated for the different illness was lower in the facility-based services than that of VHW led iCCM, and were also lower than those reported elsewhere [25]. The lower cost of the facility based service would suggest that it is a suitable approach. However VHW led iCCM was introduced to improve service coverage and timeliness in accessing care, which has been reported [10, 16].

The additional cost per child treated appropriately by the VHW led iCCM strategy was US$ 6.67 which is lower than the public health expenditure per capita for Uganda at US Dollar (US$) 21 [24].

When health expenditure per capita is used as the WTP threshold, the current study results show that the VHW iCCM strategy is cost effective for treating childhood illnesses in rural settings. Management and supervision processes have been suggested to be cost drivers [28, 29]. The rationalization of these processes has the potential to reduce implementation costs for VHW-led iCCM programmes, lowering the additional cost per child treated appropriately. VHW-led iCCM is provided in the vicinity of households in defined villages, making it more convenient and geographically accessible, resulting in improved service coverage. This demonstrates that the VHW-led iCCM in rural areas significantly supplements facility-based services for the management of childhood illness and may reduce the demands on the existing health infrastructure.

## Limitations

The study data comes from a VHW-led iCCM in a single subcounty (Bugoye subcounty). The context and VHWs, however, are typical of other rural settings in the country. As a result, the findings are comparable to similar settings in Uganda.

## Conclusion

When compared to VHW-led iCCM, health facility-based services are less expensive per child treated appropriately. The VHW-led iCCM, on the other hand, was more effective in terms of the number of children treated for malaria, pneumonia, and diarrhoea. Considering Uganda’s per capita public health expenditure as the willingness to pay threshold, VHW-led iCCM is a cost-effective strategy. VHW-led iCCM should thus be sustained and strengthened as an option to supplement health-care facility-based services for the treatment of childhood illnesses in rural settings.

## Data Availability

All data supporting our findings are contained in the paper. There are no restrictions to data sources, however, details of the full data may be accessed through Prof. Edgar Mugema Mulogo (corresponding author), Department of Community Health, Mbarara University of Science and Technology, PO Box 1410, Mbarara, Uganda, email: emulogo2000@gmail.com, Tel: + 256,772,433,508, Skype address; edgar.mulogo1.
